# Synthetic Scaffold/Dental Pulp Stem Cell (DPSC) Tissue Engineering Constructs for Bone Defect Treatment: An Animal Studies Literature Review

**DOI:** 10.3390/ijms21249765

**Published:** 2020-12-21

**Authors:** Felice Lorusso, Francesco Inchingolo, Gianna Dipalma, Francesca Postiglione, Stefania Fulle, Antonio Scarano

**Affiliations:** 1 Department of Innovative Technologies in Medicine & Dentistry, University of Chieti-Pescara, Via dei Vestini 31, 66100 Chieti, Italy; francesca-postiglione@hotmail.com (F.P.); ascarano@unich.it (A.S.); 2 Department of Interdisciplinary Medicine, University of Bari “Aldo Moro”, 70121 Bari, Italy; f.inchingolo@icloud.com; 3 Department of Basic Medical Sciences, Neurosciences and Sense Organs, University of Bari Aldo Moro, 70121 Bari, Italy; giannadipalma@tiscali.it; 4 Department of Neuroscience, Imaging and Clinical Sciences, University “G. d’Annunzio” of Chieti-Pescara, 66100 Chieti, Italy; stefania.fulle@unich.it

**Keywords:** dental pulp stem cells, synthetic scaffold, bone regeneration, tissue engineering

## Abstract

Background: Recently a greater interest in tissue engineering for the treatment of large bone defect has been reported. The aim of the present systematic review and meta-analysis was to investigate the effectiveness of dental pulp stem cells and synthetic block complexes for bone defect treatment in preclinical in vivo articles. Methods: The electronic database and manual search was conducted on Pubmed, Scopus, and EMBASE. The papers identified were submitted for risk-of-bias assessment and classified according to new bone formation, bone graft characteristics, dental pulp stem cells (DPSCs) culture passages and amount of experimental data. The meta-analysis assessment was conducted to assess new bone formation in test sites with DPSCs/synthetic blocks vs. synthetic block alone. Results: The database search identified a total of 348 papers. After the initial screening, 30 studies were included, according to the different animal models: 19 papers on rats, 3 articles on rabbits, 2 manuscripts on sheep and 4 papers on swine. The meta-analysis evaluation showed a significantly increase in new bone formation in favor of DPSCs/synthetic scaffold complexes, if compared to the control at 4 weeks (Mean Diff: 17.09%, 95% CI: 15.16–18.91%, *p* < 0.01) and at 8 weeks (Mean Diff: 14.86%, 95% CI: 1.82–27.91%, *p* < 0.01) in rats calvaria bone defects. Conclusion: The synthetic scaffolds in association of DPSCs used for the treatment of bone defects showed encouraging results of early new bone formation in preclinical animal studies and could represent a useful resource for regenerative bone augmentation procedures

## 1. Introduction

Stem cell therapies and tissue engineering have been proposed as useful strategies for the treatment of damaged tissue and bone defects [1,2,3,4,5,6,7,8]. The reconstruction of large bone defects often requires the using of biomaterials and substitutes able to provide the new regeneration and remodeling of the bone tissues due to osteoconduction, osteoinduction and osteogenesis properties [9,10]. Osteoconduction is a process correlated to the capability of a biomaterial to create the physical space-maintaining of the regenerative space, to create the stability of the blood clot in the healing period and to provide the reparative growth of the native bone [11,12,13,14,15]. Osteoinduction is a process in which the biomaterial shows the capability to stimulate the recruitment, proliferation and the differentiation of the osteoprogenitor cells, inducing new bone formation [11,16,17,18,19,20]. The osteogenesis property is typical of biomaterials self-provided by osteoprogenitors cells, such as autologous graft that represents the gold standard for bone regeneration [11,16,21]. The disadvantage of the present technique is correlated with the surgical graft donor site, which indicates the manageability of the procedure and increases the biological costs of the surgery [22,23]. The main bone substitute characteristics are biocompatibility, remodeling and complete substitution with new bone, no inflammatory evidence, cost-effectiveness, and high manageability [16,24]. Moreover, the optimal synthetic graft should present similar mechanical and physical properties to the replaced bone tissue, simulating the correct ratio of its cortical/cancellous components. Many different scaffold categories have been proposed for bone regeneration: metal graft, polymers, bioglasses and ceramics [11,16,25,26,27]. Tissue engineering for bone regeneration takes advantage of using the synthetic bone scaffolds seeded by multipotent stem cells [28]. The stem cells (SCs) represent progenitors with clonogenicity, multi-lineage differentiation and self-renewal capability [2,29,30]. In the literature, SC cells could be produced by several different oral donor tissues, such as by deciduous elements, periodontium and ligaments, dental follicle progenitor cells, apical papilla and gingiva [1,2,31]. The SCs, which are able to differentiate into osteoblasts cells, are locally determined by growth factors (GFs), physical loading and hormones [32,33,34,35,36]. Dental pulp stem cells obtained from tooth pulp tissues are able to differentiate between cells lines, such as osteoblasts, odontoblasts, endothelial cells, nerve cells, and adipocytes [37].

In the literature, dental pulp stem cells (DPSCs) have been proposed for bone defect treatment with tissue engineering due to high accessibility source of mesenchimal stem cells, high efficiency and easy extraction procedure [38,39,40]. In fact, the DPSCs can be obtained by tooth germ, deciduous and permanent teeth. Goto et al. reported that the Msh homeobox 1 (MSX1) is a key regulatory factor for DPSC osteogenic differentiation [41]. In vitro, an early increase in gene expression, such as runt-related transcription factor-2 (RUNX2), bone morphogenetic protein-2 (BMP2), alkaline phosphatase (ALPL), osteocalcin (OCN), and alkaline phosphatase activity, was demonstrated [41].

Moreover, DPSCs combined with biomaterials and bone graft reported a high regenerative capability for tissue engineering with high new bone formation and osteointegration [42,43].

The aim of the present systematic review and meta-analysis was to evaluate the synthetic scaffold and DPSC complexes for the treatment of bone defects in vivo in animal preclinical studies.

## 2. Results

### 2.1. Papers Identification and Selection

**Figure 1:**
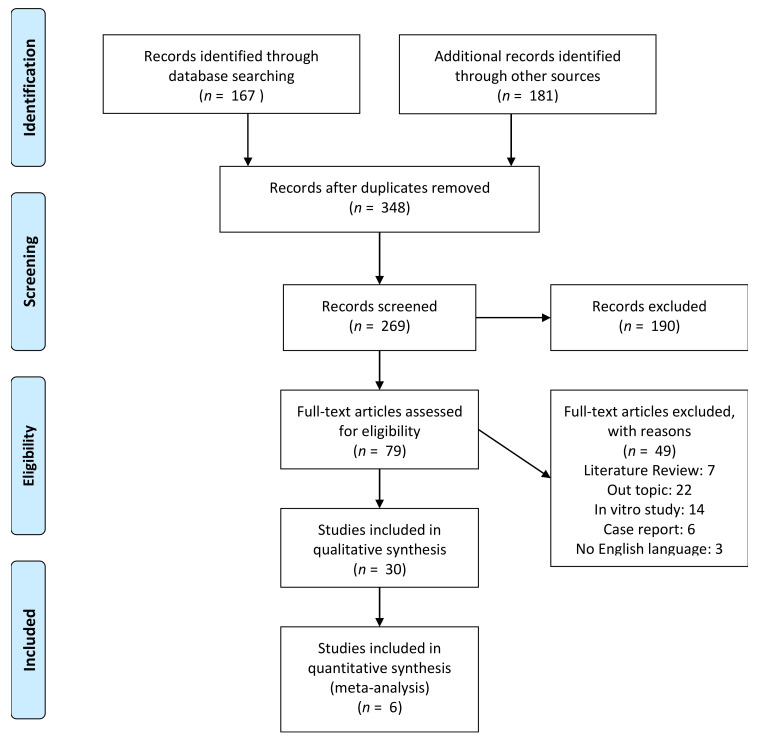
PRISMA flowchart of the studies selection for systematic review and meta-analysis.

The studies selection process was presented in Figure 1. A total of 167 matches was identified by the electronic search while a manual search provided a total of 181 records for the output list. A total of 348 papers was retrieved for the screening procedure. After a first phase of title and abstract screening, 190 papers were excluded from the list for full-text evaluation. After the eligibility evaluation, a total of 49 papers were excluded: 7 literature reviews and 22 manuscripts were off topic, 14 articles were in vitro investigations, 6 were classified as reports and 3 papers were not written in English language. A total of 30 manuscripts were included in the qualitative synthesis.

### 2.2. General Parameters

The main characteristics of the studies included are described in Table 1, Table 2, Table 3 and Table 4. The articles are categorized according the defect treated, study samples, control and tests grafted site, research outcome, and surgical follow-up time for each animal model.

The main characteristics of the bone defect model, bone substitutes and cells seeded, study outcome, and follow-up time for experiment are summarized in Table 1, Table 2, Table 3 and Table 4. The histological evaluation was performed for a total of 28 of the selected papers (93.3%) [44,45,46,47,48,49,50,51,52,53,54,55,56,57,58,59,60,61,62,63,64,65,66,67,68,69,70,71] and histomorphometry was conducted in 25 of the studies’ experiments [45,46,47,48,49,50,51,52,53,54,55,56,57,58,60,62,63,64,65,66,67,68,69,70,71] (83.3%). For a total of 18 studies (60%) micro-CT evaluation was performed [44,45,46,47,48,49,50,54,55,56,57,58,59,63,65,68,70,71] and immunohistochemistry was performed for 26 articles (86.7%) [45,46,47,48,49,50,51,52,53,54,55,56,57,58,60,61,62,63,64,65,66,67,68,69,70,71]. Scanning electron microscopy was performed for 11 studies (36.7%) [47,53,54,55,56,64,65,66,68,70,71], and one paper (3.3%) described transmission electron microscopy evaluation [70].

### 2.3. Rat Study Model

A total of 19 articles were conducted on rat models and the DPSC culture passage ranged from one to six [44,45,46,47,48,49,50,51,52,53,54,55,56,57,58,59,60,61,62]. A total of 14 papers were conducted on rat calvaria/critical size defects [46,47,48,49,50,51,53,54,55,56,58,60,62], 2 on dental alveolar defects/maxillary [44,61], 2 on dental alveolar defects/mandibular and 1 article studied tibial bone defects [45,52]. Therefore, many different bone scaffolds were used alone or in association with DPSCs, such as atelocollagen sponge [49], matrigel [44,48], puramatrix [45], dense collagen scaffold and gels [46,47,60,61,62], hemospon [57], inorganic bovine bone [54,59], 140 µm thick quartz membrane [48], β-tricalcium phosphate scaffolds (β-TCP) [52,56], composite strontium folate (SrFO)-TCP [55], hydroxiapatite/tricalcium phosphate paste (HA-TCP) [50], hydrogels [51], biodegradable polyesters [58,64], nanofibers [53].

### 2.4. Rabbit Study Model

A total of three studies were performed on rabbit models and the DPSC culture passage ranged from three to seven [65,66,67]. One article was conducted on femur diaphysis [66], one article on [67], and one study on calvaria/critical size defect [65]. The biomaterials used alone or in association with DPSCs were bonelike/tisseel mix [66], deproteinized bovine bone graft [67], polycaprolactone (PCL)–biphasic calcium phosphate (BCP) [65].

### 2.5. Sheep Study Model

A total of four studies were conducted on sheep models and the DPSC culture passage ranged from one to four [63,64]. One article was conducted on calvaria/critical-sized defects with inorganic bovine bone [63] and one paper was conducted on alveolar bone defects with the collagen/poly(L-lactide) scaffold [64].

### 2.6. Swine Study Model

A total of four studies were conducted on swine models and the DPSC culture passage ranged from three to four [68,69,70,71]. Three studies were performed on alveolar bone defects [68,69,71] and one paper on periodontal molar bone defects [70].

### 2.7. Study Risk of Bias

**Figure 2:**
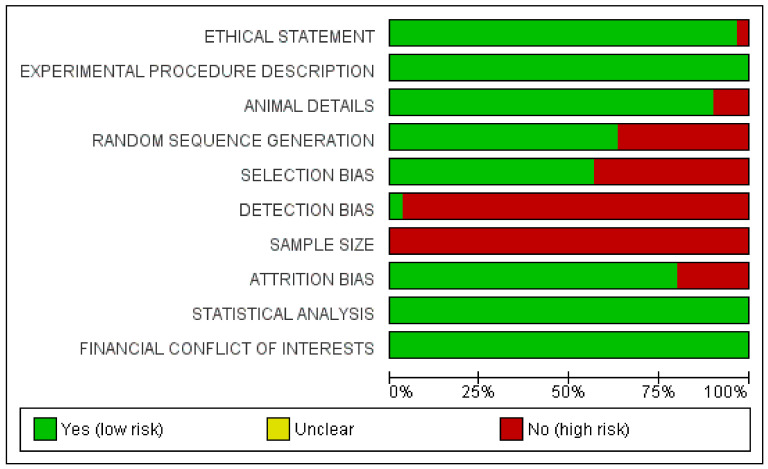
Summary of risk of bias graph of papers selected for systematic review.

**Figure 3:**
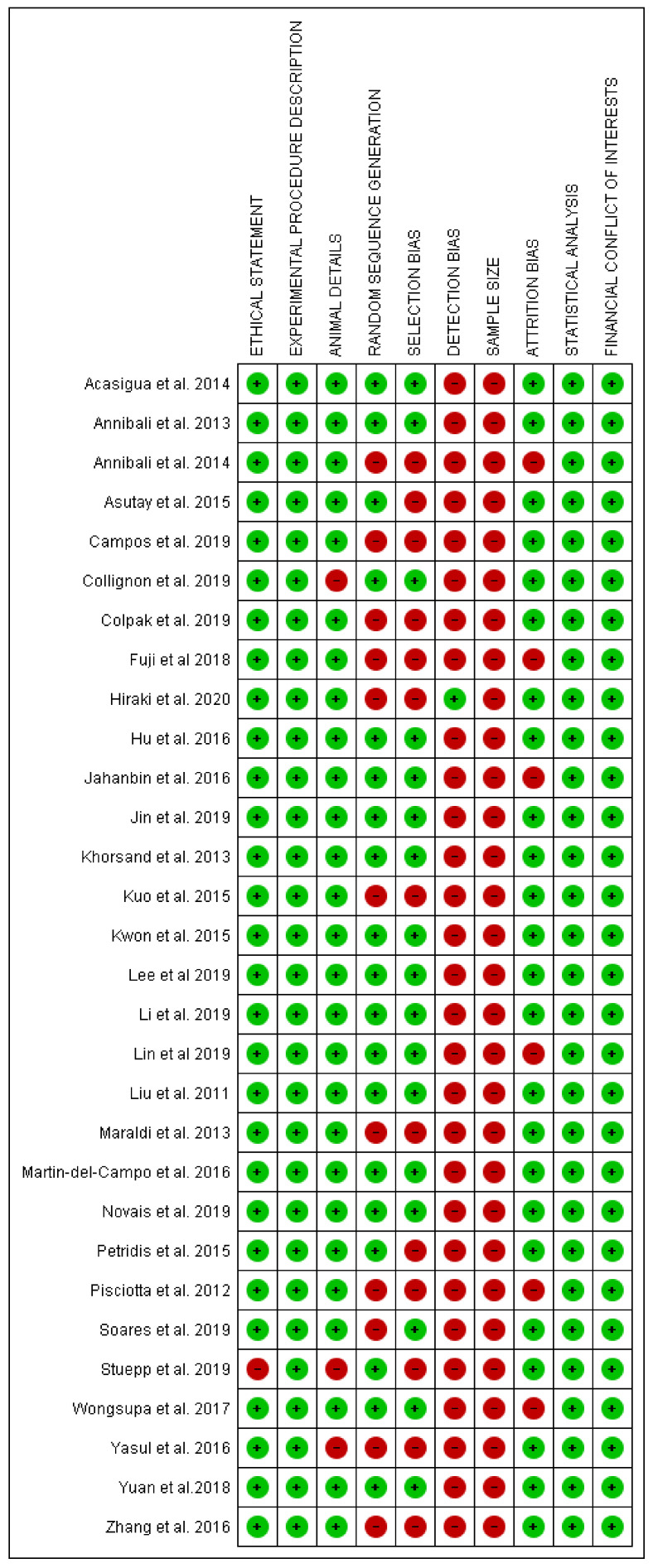
Graph of risk of bias assessment of all papers included in present systematic review.

The risk of bias evaluation was performed for all selected articles across all included articles and is presented in Figure 1. A total of 20 articles showed a low risk of bias, while the other papers were associated with high risk of bias (Figure 2 and Figure 3) [44,45,46,47,49,50,51,53,54,55,56,57,58,61,63,64,65,68,70,72].

The included papers showed a large heterogeneity related to the animal model design, experimental site and defect, methods and measurements and follow up period. The sample size calculation description represented the aspect with the highest risk, while in general the overall low-risk studies represented 66.7% of the papers included for the qualitative analysis and 83.3% of the papers included for the data meta-analysis. The longest follow-up period was conducted by Zheng et al. after 24 weeks during a study on minipigs [69]. In several included studies, the scaffold without the DPSCs represented the most-used comparative control.

### 2.8. Meta-Analysis Evaluation

After study data recording, a total of six comparative papers with histomorphometric new bone formation by synthetic bone blocks and DPSC complexes vs. scaffold only as a control group were selected. The experimental outcomes were classified according to the follow-up times: four weeks and eight weeks.

A total of six studies [46,49,51,54,58,60] performed on rats were included in the present meta-analysis investigation due to the differences between the study models adopted, control group, bone defect characteristics and study follow-up.

**Figure 4:**
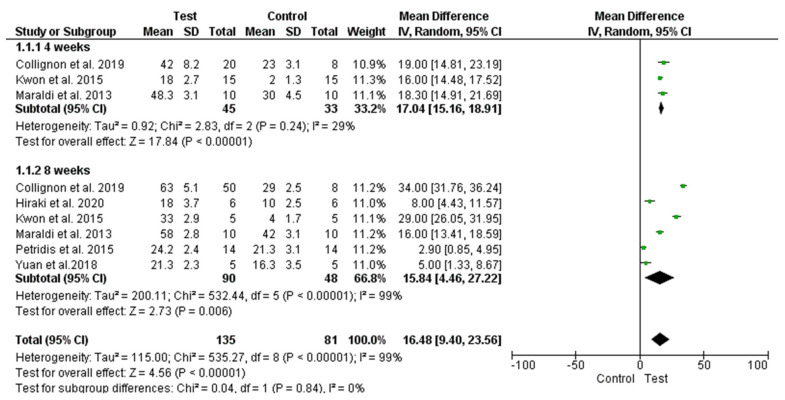
Forest plot of comparison: of new bone formation, of the DPSCs (**right**) and synthetic scaffold complexes (**left**).

The studies comparing synthetic blocks versus unfilled sites were excluded from the meta-analysis and the results are presented in Figure 4. The meta-analysis procedure demonstrated significantly higher new bone formation in the groups using DPSCs bone blocks compared to the control group. The difference appears to smoothen slightly at the longer follow-up. Moreover, a significant heterogeneity was present between the studies at 8 weeks, while half of studies presented a nonsignificant effect.

### 2.9. Articles Excluded from the Meta-Analysis

The papers screening showed a wide heterogeneity of the bone defect and animal models. The calvaria critical size defects were produced in nine studies [47,48,50,53,55,56,59,63,65], while an experiment was performed on a rabbit model and five investigations were conducted on rats. The alveolar bone defect was performed in five different studies, four of which were on rats [44,45,52,61], four on minipigs [68,69,70,71] and one on rabbits [64]. In 10 studies, the control group was represented by an empty defect [50,54,57,62,63,65,68,69,70,71], and in another study, two controls were identified with and without membranes covering the bone defect [48]. In a study, the bone defect was produced on femur diaphysis in a sheep model, while Campos et al. evaluated a complex of glass-reinforced hydroxyapatite (HA) composite with fibrin sealant [66]. At 120 days, a new bone formation of 67.9 ± 3.9% and 77.5 ± 3.2%, respectively, for scaffold complexes without and with DPSCs was achieved. The shortest follow-up period was performed by Acasigua et al. on murine calvaria critical-sized defect treated with DPSC/polylactic-co-glycolic acid nanofiber complexes after 6 days [34]. A higher gain in bone regeneration was reported for the DPSC complexes when compared to the scaffold alone. Annibali et al. reported the wider sample size of the studies selected with a total of 150 sites and 75 rats [56], while two different stem cell populations were studied in association with granular deproteinized bovine bone. Higher new bone formation was reported with periosteal stem cell complexes if compared to DPSC with granular deproteinized bovine bone and scaffold alone conditions. Pisciotta et al. performed research on rat parietal bone critical-sized defects with DPSCs seeded on collagen scaffold compared to empty defects [62]. Soares et al. reported on murine tibial bone defects treated with hemospon/8% aloe vera with and without DPSC complexes and increased new bone formation when compared to the empty defects [57].

## 3. Discussion

The aim of the present study was to investigate, through systematic review and meta-analysis, the in vivo outcome of synthetic scaffolds and DPSC complexes for bone regeneration on animal models. The paper screening identified a large heterogeneity of animal model design methods and follow up periods. The interspecies characteristics regarding the healing patterns and periods of bone reparative processes probably represent a factor for comparative evaluation between the selected studies in the same way of the differences regarding DPSCs isolation and differentiation protocols. The papers’ selection for the review included a total of five different species, such as rabbit, murine, swine, and sheep, while the most represented was the rat model. These species are characterized by specific properties that should be considered for a translational comparison to the human model [73,74,75]. The adoption of a small-size animal, such as murine or rabbits is often associated with an easier surgical procedure, shorter healing period of the bone defect and lower management cost of the research [76,77,78]. The higher quantity of papers studied the rat model, while the most represented defect was the calvaria critical size defect. The rat critical size defect was defined as an 8 mm diameter osteotomy [79] and could be performed according to a drilling procedure [80,81,82], trephine approach [79], ultrasonic tips [83] or elevator technique [79]. Parietal bone defects have been proposed, according to a craniofacial fibrous nonunion research model, as being able to provide a standardized technique for bone healing and biomaterial osteointegration [79,84]. In almost all studies selected, the DPSC treatment was characterized by a higher percentage of new bone formation compared to the control site after 4- and 8-week healing periods. The meta-analysis was not performed on rabbits, swine and sheep studies, according to the wide heterogenicity of the bone defects model, study design and healing period. Additionally, the local anatomy of the defect site could represent a key factor: calvaria critical size defects have been proposed as a refined and more reproducible model for bone regeneration for both craniofacial and long-bone repair [50,51]. The adoption of a dental alveolar bone defect could be initiated for comparison through wide local anatomical and bone density differences present between the two jaws and also the posterior and anterior sites [85].

### 3.1. Synthetic Scaffolds

In the studies included for the qualitative analysis, wide categories of scaffolds and bone substitutes were evaluated, such as fibrin biomaterials [86,87], hydrogels, nanofibers [53], polyethylene glycol hydrogels [88], beta tricalcium phosphate (βTCP) [89,90] hydroxyapatite and bioglass derivates [91,92], deproteinized heterologous bone [93], absorbable gelatin sponges [94] and collagen scaffolds [95], have been studied in vivo in combination with the DPSCs in the present review. These studies proved the several types of biomaterials that are currently under investigation for bone tissue regeneration with DPSCs. The selected papers showed, with all biomaterials, a good integration of the scaffold associated with new bone formation with or without DPSCs. The capacity of adaptation, the manageability, three-dimensionality, osteoconductive, bacteriostatic, and total reabsorption of the scaffold could probably represent a key factor for bone regeneration and should be preferred for clinical use [92,96].

The advantage related to scaffold use is determined by the physical space-maintainig capability of the regenerative area, the sustaining of the three-dimensional structure, and the support of the healing processes through the growth factor and host cellular osteogenic response [97,98].

The main scaffold properties should provide mechanical stability, good compatibility and progressive substitution with the new bone according to the tissue remodeling [51].

The identification of a suitable scaffold for tissue engineering is a key factor, due to its interaction with DPSCs and their differentiation and proliferation induction. Moreover, the scaffold’s three-dimensional microstructure, and micropore presence, is essential for DPSC adhesion and proliferation, also allowing nutrient support and oxygen diffusion [96,99].

In the present review, almost all scaffolds and bone substitutes presented satisfactory results in terms of DPSC tissue engineering potential and new bone regeneration.

### 3.2. Limitations of the Research

The present investigation presented some limitations. In fact, the level of evidence is determined by the quality of the articles. Most of the studies selected presented a low risk of bias (19 on 33 articles). The DPSC/bone scaffold complexes represent an innovative model not previously evaluated in humans, and this could be a factor for the translational value of the research outcome. Additionally, the wide heterogenicity of study designs, animal models, bone defects, biomaterials properties, control sites (empty defects, autologous graft, scaffold alone), and follow-up periods could represent factors that could indicate the comparative evaluation.

## 4. Materials and Methods

### 4.1. Database Search

The present investigation, systematic review and meta-analysis was conducted according the PRISMA guidelines.

The electronic database search was conducted on PubMed, Scopus, and EMBASE (accesed on 4 October 2020), according the following Boolean search paradigm: “Bone Regeneration AND Dental Pulp Stem Cell AND Animal studies”. The manuscripts identified were limited at only scaffold bone graft and DPSCs on in vivo animal studies. Moreover, a manual search was also performed to find additional eligible articles not identified by the electronic search.

### 4.2. Inclusion Criteria

For the systematic review, the comparative animal studies were included with no restrictions regarding the species, bone defects and types of synthetic blocks. The reviews, letters to the editor, clinical reports, case series, and in vitro studies were excluded from the present investigation.

### 4.3. Selection of the Studies

The identification of the animal studies eligible for the review was conducted independently by two reviewers evaluating the manuscript title and abstract. The full text was evaluated in this first phase in the case of the abstract being unavailable. Only the papers written in English were considered for the evaluation. The studies that did not satisfy the inclusion criteria were excluded from the review. The complete full text of all manuscript was than obtained and evaluated. The excluded articles were also categorized and the reasons for exclusion from the investigation were recorded.

### 4.4. Data Extraction

The data obtained by the included articles were extracted and analyzed independently by the two authors (F.L., A.S.), following a specially designed data-collection form, which ensured the systematic recording of data. The aim was to quantitatively and qualitatively evaluate the outcome of the synthetic biomaterial blocks in bone regenerative procedures. The primary outcomes were the percentage of new bone formation and the percentage of residual bone. The secondary outcomes were the bone density at radiographic analysis, the soft tissue health, and the postoperative complications. Other data were the sample size, the gender, the duration of follow-up, the bone defect size and location, and the type of biomaterial used in the test and in the control group.

### 4.5. Risk of Bias

The risk of bias assessment was conducted according the Animals in Research: Reporting In Vivo Experiments (ARRIVE) guidelines for animal studies. The criteria for the risk of bias assessment were ethical statement, description of experimental procedure, animals details, randomization sequence, selection bias, detection bias, sample size evaluation, attrition bias, statistical evaluation and conflict of interests. The risk of bias criteria was categorized as adequate, unclear, or inadequate. A low-risk study was considered as having a value of at least 7/10 appropriate parameters. Otherwise, the studies were classified as high risk. The evaluation was conducted by the dedicated software package RevMan 5.5 (The Nordic Cochrane Centre, The Cochrane Collaboration, Copenhagen, 2014).

### 4.6. Review and Meta-Analysis Criteria

The study data were collected by a special designed database with the software package Excel (Microsoft, Redmond WA USA). For the meta-analysis, the comparative investigation between DPSCs/synthetic block vs. scaffold alone was considered. The manuscripts that did not submit the criteria were not considered, while only the papers with similar comparative evaluations reporting the same outcome measures were included. The mean differences were considered for continuous data if at least four studies were considered, and if there were less than four studies, a fixed-effects model was adopted. On split model research, a null intraclass correlation coefficient was considered. The meta-analysis was conducted by the software package RevMan 5.5 (The Nordic Cochrane Centre, The Cochrane Collaboration, Copenhagen, 2014). The outcome variable evaluated was the percentage of new bone formation in the test and control groups by histomorphometric evaluation.

## 5. Conclusions

The preclinical studies’ evidence showed that DPSCs associated with synthetic bone scaffolds presented potential efficacy for stem cell treatment of bone defects. The encouraging effectiveness of early new bone formation in animal models should be considered for innovative bone defect treatment protocols in future trials on human models due to the easy availability and expansion of the stem cells

## Figures and Tables

**Table 1:** General features of the nineteen studies performed on rats. (BMP-2: recombinant human bone morphogenetic protein; 2PGA: polylactic-co-glycolic acid; TCP: Tricalcium phosphate; SHED:10^5^ DPSCs cells/atelocollagen sponge; SHED-CM: 40 × 25 μL Culture medium/atelocollagen sponge; Memb: Membrane ADSC: Adipose stem cells; cBMSCs: canine bone marrow stem cells; cDPSCS: canine dental pulp stem cells; pDPSCS: puppy dental pulp stem cells; PRP: platelet rich plasma; Hist: Histology; Histom: Histomorphometry; IMM: immunohistochemistry; MCT: Micro CT; SEM: Scanning electron Microscopy; CONF MICRO: confocal microscopy; NBF: New bone formation.)

Authors	Journal	Year	Defect	Samples	Test	Control	DPSCS Expansion	Analysis Methods	Follow Up	NBF Test	NBF CTR
Hiraki et al. [49]	Oral Dis	2020	Calvaria/critical-sized defect	18 animals/18 sites	(1) SHED (2) SHED-CM	serum-free α- Minimum Essential Medium Eagle (25 μL/atelocollagen sponge).	6 Passage	HIST, HISTOM, MCT, IMM,	8 weeks	(1) 18 ± 3.7%; (1) 30 ± 4.1%	10 ± 2.5%
Lin et al. [44]	J Endod.	2019	dental alveolar defects/maxillary	10 animals	DPSC/Matrigel	Matrigel	6 Passage	HIST, MCT	2 weeks	-	-
Jin et al. [45]	Artif Cells Nanomed Biotechnol	2019	dental alveolar defects/mandibular	15 animals/15 sites	(1) DPSC/0.2% Puramatrix; (2) ADSC/0.2% Puramatrix	(1) 0.2% Puramatrix	2–5 Passage	HIST, HISTOM, MCT, IMM,	6 weeks	-	-
Collignon et al. [46]	Stem Cells.	2019	Calvaria/critical-sized defect	38 animals/176 sites	(1) dense collagen scaffold seeded with fluorescent T-mDPSCs; (2) noncellularized dense collagen scaffold	Empty defect	3 Passage	HIST, HISTOM, MCT, IMM,	2, 4, 8, and 12 weeks	30 days: (1) 42%± 8.2%; (2) 23% ± 3.1. 60 days: (1) 63%; ± 5.1; (2) 32% ± 2.9 90 days: (1) 73.5% ± 4.6; (2) 39.7% ± 2.3	30 days: Control: 22% ± 2.1 60 days: Control: 29% ± 2.5 90 days: Control: 35% ± 2.3
Novais et al. [47]	Stem Cells Transl Med	2019	Calvaria/critical-sized defect	30 animals/60 sites	(1) DPSC/Plastically compressed collagen gels (Hypoxia priming); (2) DPSC/Plastically compressed collagen gels (FGF-2 priming)	Plastically compressed collagen gels (no priming)	3–4 Passage	HIST, HISTOM, MCT, IMM, SEM	14 days, 2 months,	14 days: (1) 3 ± 1.8 (2) 6 ± 3.5% 2 months: (1) 28 ± 3.9 (2) 30 ± 3.7%	14 days: 2 ± 2.4%, 2 months: 20 ± 3.2
Soares et al. [57]	J Appl Oral Sci	2019	Tibial bone defects	75 animals/75 defects	(1) Hemospon (2) Hemospon 8% Aloe vera; (3) Hemospon hDPSCs (4) (3) Hemospon 8% Aloe vera/hDPSCs	Empty defect	5 Passage	HIST, HISTOM, MCT, IMM,	1, 2 3 weeks	1 week, (1) 2.1 ± 0.1; (2) 2.1 ± 0.2; (3) 1.5± 0.3. (4) 2.6 ± 0.4, 2 week, (1) 1.6 ± 0.2; (2) 1.9 ± 0.2; (3) 2.4± 0.4. (4) 2.6 ± 0.5, 3 week, (1) 2.4 ± 0.2; (2) 2.4 ± 0.3; (3) 2.4± 0.5. (4) 2.5 ± 0.3,	1 week, (1) 1.6± 0.4; 2 week, (1) 2.3 ± 0.2; 3 week, (1) 1.8± 0.5;
Yuan et al. [54]	Int J Mol Med	2018	Calvaria/critical-sized defect	40 animals	(1) BO group, (Bio‑Oss); (2) DPSC/BO group, (2)DPSCs+ Bio‑Oss; (3) DPSC/BO/Aspirin group.	Empty defect	3 Passage	HIST, HISTOM, MCT, IMM, SEM	8, 12 weeks	8 weeks (1) 16.3 ± 3.5; (2) 21.3 ± 2.3 (3) 27.9 ± 1.5; 12 weeks (1) 21 ± 2.6; (2) 36.8 ± 3.3 (3) 59.7 ± 4.3	8 weeks 5.6 ± 3.1; 12 weeks 15.4 ± 2.8
Yasui et al. [48]	J DentRes	2016	Calvaria/critical-sized defect	6 animals/12 sites	(1) DPSC LNGFR+ THY+ cells; (2) DPSC LNGFR (low+) THY+ cells	(1) Memb+; (2) No Memb	1–5 Passage	HIST, HISTOM, MCT, IMM, CONF MICRO	2, 4 weeks	2 weeks (1) 30.6 ± 4.7%; (2) 52.8 ± 5.9%; 4 weeks (1) 10.5 ± 4.2%; (2) 19.7 ± 3.1%;	2 weeks (1) 1.2 ± 0.7%; (2)1.8 ± 0.7%; 4 weeks (1) 0.5 ± 0.3%; (2) 0.7 ± 0.4%;
Zhang et al. [52]	Tissue Eng Part A	2016	Mandible defect	5 animals/24 sites	(1) DPSC-high, CH scaffolds; (2) DPSC-low, CL scaffolds; (3) acellular scaffolds (SA); (4) acellular scaffolds supplemented with 4 μg	-	-	HIST, HISTOM, IMM,	3, 6 weeks	(1) 3 weeks: 0.5 ± 0.3%, 6 weeks: 0.8 ± 0.4% (2) 3 weeks: 0.3 ± 0.1%, 6 weeks: 0.2 ± 0.2% (3) 3 weeks: 0.4 ± 0.2%, 6 weeks: 0.6 ± 0.3% (4) 3 weeks: 22 ± 3%, 6 weeks: 21 ± 0.5%,	-
Martin-del-Campo et al. [55]	Biomater. Sci	2016	Calvaria/critical-sized defect	18 animals/36 sites	DPSC/Strontium folate (SrFO) TCP	DPSC/TCP composite	3 Passage	HIST, HISTOM, MCT, IMM, SEM	4, 12, 20 weeks	4 weeks (1) 51.2 ± 3.3; 12 weeks (1) 82.3 ± 2.7; 20 weeks (1) 86.9 ± 2.5;	4 weeks (2) 40.2 ±2.1; 12 weeks (2) 55.5 ± 2.2; 20 weeks (1) 56.8 ± 5.2;
Jahanbin et al. [61]	J OralMaxillofac Surg	2016	dental alveolar defects/maxillary	60 animals	Group 1: collagen+ iliac bone graft 1 monthGroup 2: collagen + iliac graft 2 monthsGroup 3: scaffold/DPSC 1 monthsGroup 4: scaffold/DPSC 2 monthsGroup 5: scaffold 1 monthGroup 6: scaffold after 2 months	-	-	HIST, IMM,	4, 8 weeks	Group 1: 50.0% ± 1.3Group 2: 62.5% ± 2.1Group 3: 16.7% ± 2.4Group 4:40.0% ±2.1Group 5: 0% Group 6: 0%	-
Asutay et al. [50]	Arch Oral Biol	2015	Calvaria/critical-sized defect	15 animals/30 sites	(1) HA/TCP paste; (2) HA/TCP paste/DPSC	Empty defect	-	HIST, HISTOM, MCT, IMM,	8 weeks	-	-
Petridis et al. [51]	J Craniomaxillofac Surg	2015	Calvaria/critical-sized defect	30 animals/42 sites	(1) DPSC/Hydrogel scaffold; (2) Hydrogel scaffold	Empty defect	2 Passage	HIST, HISTOM, IMM,	8 weeks	(1) 21.3 ± 2.4 (2) 34.2 ± 3.1%	20 ± 2.2%
Kwon et al. [58]	Sci Rep	2015	Calvaria/critical-sized defect	30 animals/30 defects	DPSCs/biodegradable polyesters (PLGC)	biodegradable polyesters (PLGC)	>5 Passage	HIST, HISTOM, MCT, IMM,	4, 8, and 12 weeks	4 weeks: 18% ± 2.7 8 weeks: 33% ± 2.9, 12 weeks: 58% ± 2.6	4 weeks: 2% ± 1.3 8 weeks: 4% ± 1.7, 12 weeks: 8% ± 1.5
Acasigua et al. [53]	Curr Stem Cell Res Ther	2014	Calvaria/critical-sized defect	20 animals/20 sites	I–sham; II–without cells; III PGA nanofibers/DPSC; IV–PGA nanofibers/DPSC 13 d medium	-	5 Passage	HIST, HISTOM, SEM, IMM,	6 days	8.13 ± 3.12%, 9.39 ± 2.55%, 10.7 ±3.22% and 17 ± 4.31% in groups I, II, III and IV, respectively	-
Annibali et al. [59]	J Biomed Mater Res B ApplBiomater	2014	Calvaria/critical-sized defect	16 animals/32 sites	b-TCP (b); b-TCP/DPSC (b/C); GDPB (G) andGDPB/DPSC	-	-	HIST, MCT	4, 8, and 12 weeks	-	-
Annibali et al. [56]	J Craniofac Surg.	2013	Calvaria/critical-sized defect	75 animals/150 defects	(1) DPSC/Granular deproteinized bovine bone, (2) PeriostealStem Cells PESC/Granular deproteinized bovine bone	Granular deproteinized bovine bone	-	HIST, HISTOM, MCT, IMM, SEM	1, 2, 4 8 weeks	1 week, (1) 6.7 ± 2.9; (2) 8.3 ± 3.1; 2 weeks: (1) 6.1 ± 1.7; (2) 12.1 ± 2.4; 4 weeks (1) 6.1 ± 1.7; (2) 12.3 ± 2.6, 8 weeks (1) 8.9 ± 3.8; (2) 15.4 ± 2.8	1 week, 5.3 ± 2.3; 2 weeks: 10.8 ± 2.4; 4 weeks 15.2 ± 4.8, 8 weeks 22.3 ±4.5%
Maraldi et al. [60]	Stem Cell Res Ther	2013	Calvaria/critical-sized defect	30 animals/60 sites	(1) DPSC/collagen scaffolds; (2) AFSC/collagen scaffolds	collagen scaffolds	3 Passage	HIST, HISTOM, CONF MICRO, RX, IMM,	4, 8 weeks	4 weeks: (1) 48.3 ± 3.1% (2) 52.3 ± 1.9; 8 weeks (1) 58 ± 2.8; (2) 71.1 ± 3.3%	4 weeks, 30 ± 4.5%; 8 weeks (1) 42 ± 3.1%
Pisciotta et al. [62]	PLoS One	2012	Calvaria/critical-sized defect	10 animals/10 sites	DPSCs/collagen scaffold (1) FCS serum (2) HS serum	Empty defect	5 Passage	HIST, HISTOM, IMM,	40 days	FCS: 51.3% ± 3.3; HS: 68.2 ± 4.3	Control: 42 ±3.5%

**Table 2:** General features of the three studies performed on rabbits. (cBMSCs: canine bone marrow stem cells; cDPSCS: canine dental pulp stem cells; pDPSCS: puppy dental pulp stem cells; PRP: platelet rich plasma; Hist: Histology; Histom: Histomorphometry; IMM: immunohistochemistry; MCT: Micro CT; SEM: Scanning electron microscopy; CONF MICRO: confocal microscopy; NBF: New bone formation.)

Authors	Journal	Year	Defect	Samples	Test	Control	DPSCS Expansion	Analysis Methods	Follow Up	NBF Test	NBF CTR
Campos et al. [66]	Regen Biomater	2019	Femur Diaphysis	12 animals/60 sites	(1) Bonelike/Tisseel LyoV; (2) DPSC/Bonelike/Tisseel LyoV	Empty defect	5–7 Passage	HIST, HISTOM, SEM, IMM,	30, 60 and 120 days	30 days: (1) 13.1 ± 2.9% (2) 15.2 ± 2.5%; 60 days: (1) 48.5 ± 3.7% (2)59.4 ± 3.5%;120 days: (1) 67.9 ± 3.9%, (2) (77.5 ± 3.2%)	30 days: 8.6% ± 2.3; 60 days: 45.3%± 2.5120 days: 62.6± 3.4%,
Çolpak et al. [67]	J Stomatol Oral Maxillofac Surg	2019	Iliac crest peri implant defect	5 animals/60 sites	(1) Implant without graft; (2) Implant+ DPSCs/deproteinized bovine bonegraft (DBBG)	Empty defect	4–6 Passage	HIST, HISTOM, IMM	3, 6 weeks	3 weeks (1)11.3 ± 2.3 mm, (2) 14.6 ± 3.2 mm; 6 weeks (1)18.7 ± 3.1 mm, (2) 29.3 ± 3.4 mm;	3 weeks: 2.8 ± 2.4 6 weeks: 4.1 ± 2.5%
Wongsupa et al. [65]	J Mater Sci Mater Med	2017	Calvaria/critical-sized defect	18 animals/36 sites	(1) hDPSCs seededin (PCL)–biphasic calcium phosphate (BCP)with the modified melt stretching and multilayer deposition(mMSMD) scaffolds; (2) mMSMD PCL-BCPscaffolds alone, autogenous bone	Empty defect	3–5 Passage	HIST, HISTOM, MCT, IMM, SEM	4 weeks	-	-

**Table 3:** General features of the two studies performed on sheep. (cBMSCs: canine bone marrow stem cells; cDPSCS: canine dental pulp stem cells; pDPSCS: puppy dental pulp stem cells; PRP: platelet rich plasma; Hist: Histology; Histom: Histomorphometry; IMM: immunohistochemistry; MCT: Micro CT; SEM: Scanning electron microscopy; CONF MICRO: confocal microscopy; NBF: New bone formation.)

Authors	Journal	Year	Defect	Samples	Test	Control	DPSCS Expansion	Analysis Methods	Follow Up	NBF Test	NBF CTR
Lee et al. [63]	Int J Mol Sci	2019	Calvaria/critical-sized defect	12 animals/48 sites	(1) Bio-Oss; (2) BMSCs/Bio-Oss; (3) DPSCs/Bio-Oss	Empty defect	2–4 Passage	HIST, HISTOM, MCT, IMM	3 weeks	(1) Bio-Oss: 17.2 ± 1.9%; (2) BMSCs/Bio-Oss: 22.6± 3.2; (3) DPSCs/Bio-Oss 23.4 ±5.7%	Control: 9.9 ± 2.6%)
Liu et al. [64]	TissueEng Part A	2011	Alveolar bone defect	36 animals/36 sites	(1) nHAC/PLA, (2) nHAC/PLA+ rhBMP-(2, 3) nHAC/PLA +DPSCs (4) nHAC/PLA+ DPSCs + rhBMP-2	Autologous	1 Passage	HIST, HISTOM, SEM, RX, IMM,	12 weeks	12 weeks: 21 ± 2.1%; (2) 24.4 ± 3.1%; (3) 34.1± 2.8% (4) 60.1± 3.2; (5) 54± 4.2%	12 weeks: 0%

**Table 4:** General features of the four studies performed on swine. (cBMSCs: canine bone marrow stem cells; cDPSCS: canine dental pulp stem cells; pDPSCS: puppy dental pulp stem cells; PRP: platelet rich plasma; Hist: Histology; Histom: Histomorphometry; IMM: immunohistochemistry; MCT: Micro CT; SEM: Scanning electron Microscopy; CONF MICRO: confocal microscopy; CSD: calcium sulfate dehydrate; CSH: calcium solfate hydrate; ACP: amorphous calcium phosphate; β-TCP: β-tricalcium phosphates; NBF: New bone formation.)

Authors	Journal	Year	Defect	Samples	Test	Control	DPSCS Expansion	Analysis Methods	Follow Up	NBF Test	NBF CTR
Li et al. [32]	TissueEng Regen Med	2019	Alveolar bone defect	6 animals/48 sites	(1) DPSCs/beta tricalcic Phosphate b-TCP. (2) b-TCP	Empty defect	3 Passage	HIST, HISTOM, MCT, IMM, SEM	12 weeks	-	-
Hu et al. [39]	Stem Cell Res Ther	2016	Periodontal molar bone defects	12 animals/48 defects	(1) hDPSC injection group, (2) hDPSC sheets	Empty defect	3–4 Passage	HIST, HISTOM, MCT, IMM, SEM, TEM	12 weeks	12 weeks (1) 10.5 ± 5.2; (2) 16.3 ± 4.4	12 weeks (1) 5.3.5 ± 2.1;
Kuo et al. [71]	Mater Sci Eng C Mater Biol Appl	2015	Dental alveolar defects/mandibular	12 animals/24 sites	CSD, α-CSH/ACP, and CSD/β-TCP (with/without DPSCs); (CSD), (α-CSH/ACP), and CSD/(β-TCP)	Empty defect (with/without DPSCs)	-	HIST, HISTOM, MCT, IMM, SEM	8 weeks	(1) CaSO4 33.9 ± 9.9; CaSO4/DPSC 69.7± 4.9; (2) α-CaSO4·0.5H2O/ACP 61.7± 2.3; DPSC/α-CaSO4·0.5H2O/ACP 70.5 ± 6.6; (3) CaSO4·2H2O/β-TCP 44.5± 2.9; DPSC/CaSO4·2H2O/β-TCP 57.1 ± 4.1	Empty defect: 27.0 ± 9.5; Empty defect/DPSC: 24.3 ± 5.6
Zheng et al. [69]	J Dent Res.	2009	Dental alveolar defects/mandibular	16 animals/22 sites	(1) Beta-TCP/DPSC; (2) Beta-TCP	Empty defect	3–4 Passage	HIST, HISTOM, IMM,	2,4, 24 weeks	24 weeks: (1) 83.1 ± 5.75%; (2) 52.2 ± 4.54%	24 weeks: 28.4 ± 2.79%
